# Low-level viraemia during treatment with darunavir/r monotherapy versus DRV/r + 2NRTIs in the MONET trial

**DOI:** 10.1186/1758-2652-13-S4-O19

**Published:** 2010-11-08

**Authors:** N Clumeck, JR Arribas, P Pulick, G Fätkenheuer, A Hill, Y Van Delft, C Moecklinghoff

**Affiliations:** 1 CHU Saint-Pierre / Maladies Infectieuses, Brussels, Belgium; 2 Hospital la Paz, Madrid, Spain; 3 Hospital for Infectious Diseases, Warsaw, Poland; 4 Med. Einrichtungen der Universität Köln, Klinik I für Innere Medizin, Köln, Germany; 5 Pharmacology Research Laboratories, Liverpool University, Liverpool, UK; 6 Janssen-Cilag, Tilburg, Netherlands; 7 Janssen-Cilag, Neuss, Germany

## Background

Patients with HIV RNA suppression below 50 copies/mL may still have HIV RNA detectable by more sensitive PCR assay techniques.

## Methods

In the MONET trial, 256 patients with HIV RNA <50 copies/mL on current HAART, and no history of virological failure, switched to DRV/r 800/100 mg once daily, either as monotherapy (n=127) or with 2NRTI (n=129). HIV RNA was evaluated by the Roche Amplicor Ultrasensitive assay (lower detection limit=50 copies/mL), for all patient visits to Week 96. With this assay, "Optical Density=background" was used to assess whether HIV RNA was detectable or undetectable below 50 copies/mL.

## Results

Patients were 81% male, 91% Caucasian, and had median baseline CD4 count of 575 cells/uL. At the baseline visit, the percentage of patients with HIV RNA undetectable below 50 copies/mL (OD=background) was 80% in the DRV/r mono arm and 79% in the DRV/r + 2NRTI arm. The percentage with HIV RNA at different levels at the Week 96 visit is shown in Table 1 (observed data analysis)

**Table 1:** 

HIV RNA	DRV/r mono (n=105)	DRV/r + 2NRTIs (n=114
HIV RNA <50, OD = background	79.0%	80.7%

HIV RNA <50, detectable	17.1%	14.9%

HIV RNA 50-400 copies/mL	2.9%	3.5%

HIV RNA <400 copies/mL	1.0%	0.9%

		

Including all samples from patient visits from Week 4 to Week 96, HIV RNA was above 50 copies/mL in 69/1009 samples in the DRV/r monotherapy arm (50-400: 84%, 400-1000: 12%, >1000: 4%) and 47/1051 samples in the DRV/r + 2NRTI arm (50-400: 83%, 400-1000: 8.5%, >1000: 8.5%).

## Conclusions

In this study for patients with HIV RNA <50 copies/mL at baseline, switching to DRV/r monotherapy showed similar levels of HIV RNA suppression to DRV/r + 2NRTIs, using more sensitive PCR assay techniques.

